# Knowledge, attitudes, and practices toward avian influenza among free-grazing duck farmers in Central Thailand: An analytical cross-sectional study

**DOI:** 10.14202/vetworld.2026.97-110

**Published:** 2026-01-08

**Authors:** Supanat Boonyapisitsopa, Kamonpan Charoenkul, Napawan Bunpapong, Supassama Chaiyawong, Chanakarn Nasamran, Kannika Thammasutti, Chutarat Saengkul, Somsak Pakpinyo, Kanokwan Suwannarong, Alongkorn Amonsin

**Affiliations:** 1 Department of Veterinary Public Health, Chulalongkorn University, Bangkok, Thailand; 2 Center of Excellence for Emerging and Re-emerging Infectious Diseases in Animals, Chulalongkorn University, Bangkok, Thailand; 3 Nakhon Sawan Campus, Mahidol University, Nakhon Sawan, Thailand; 4 SUPA71 Co., Ltd., Bangkok, Thailand

**Keywords:** attitude, avian influenza, free-grazing ducks, knowledge, One Health, practice, public health, Thailand

## Abstract

**Background and Aim:**

Free-grazing duck (FGD) production systems play a vital economic role in Thailand but are also recognized as potential sources and amplifiers of avian influenza (AI) viruses at the human–animal–environment interface. Understanding the knowledge, attitudes, and practices (KAP) of individuals involved in FGD production is crucial for effective prevention and control of AI. This study aimed to assess AI-related KAP levels among FGD farmers and related workers in central Thailand and to identify demographic, occupational, and behavioral factors linked to these KAP outcomes.

**Materials and Methods:**

An analytical cross-sectional survey was conducted from January to May 2023, involving 101 participants working in FGD production systems across Ayutthaya, Suphan Buri, and Nakhon Sawan provinces. Data were obtained through face-to-face interviews using a structured, expert-validated questionnaire that covered socio-demographic details, animal exposure, vaccination history, and AI-related knowledge, attitudes, and practices. KAP scores were determined using standardized scoring criteria. The relationships between KAP scores and explanatory variables were analyzed using simple and multiple linear regression.

**Results:**

The average knowledge score was 8.65 ± 2.39 (out of 12), the average attitude score was 3.63 ± 0.36 (out of 5), and the average practice score was 3.17 ± 0.38 (out of 5). Overall, 58.4% of participants demonstrated good knowledge, 66.3% exhibited positive attitudes, and 38.6% reported good preventive practices against AI. Knowledge scores were significantly linked to daily working hours with FGDs, contact with other animals, and influenza vaccination history. Positive attitudes were significantly influenced by educational level and occupation, while good practices were associated with higher education, type of FGD production system, animal contact, and vaccination during poultry work. Moderate positive correlations were observed between knowledge and attitude scores and between attitude and practice scores.

**Conclusion:**

This study offers the first comprehensive assessment of KAP regarding AI among FGD farmers in Thailand. Although knowledge and attitudes about AI were generally adequate, preventive measures were relatively inadequate. Improving targeted public health education, increasing vaccination awareness, and implementing One Health–based biosecurity measures are recommended to boost AI prevention and readiness in FGD production systems.

## INTRODUCTION

Avian influenza (AI) is a viral zoonotic disease primarily transmitted from birds to humans and represents a major public health issue. Over the last two decades, AI has gained increased global attention due to the rising number of interspecies transmissions of AI viruses (AIVs). Since the first human case was reported in Hong Kong in 1997, the highly pathogenic AI (HPAI) subtype H5N1 (HPAI-H5N1) has resulted in 992 laboratory-confirmed human cases and 476 deaths worldwide as of November 2025 [1]. AIVs cause severe respiratory illness in poultry, including chickens, ducks, turkeys, quail, and other bird species. Importantly, HPAI-H5N1 viruses can infect a wide range of mammals, such as humans, dogs, cats, tigers, and cattle, often with fatal outcomes [2, 3]. In Thailand, multiple HPAI-H5N1 outbreaks have been reported since 2004, causing significant poultry deaths and severe economic losses across commercial farms, backyard flocks, live-poultry markets, and free-grazing duck (FGD). Although no poultry outbreaks have been reported since the last epidemic wave in 2008, several studies have shown ongoing circulation of low-pathogenic AI (LPAI) viruses among poultry and wild birds in Thailand [4–6].

FGDs serve as a key reservoir for AIVs. These layer ducks are raised extensively in rice paddies and often move across large areas in search of food, which increases the potential for virus spread. FGDs frequently share habitats with wild birds and backyard poultry, creating opportunities for interspecies transmission and viral dissemination. Additionally, the coexistence of multiple AIV subtypes in FGDs may promote viral reassortment. Previous studies in Thailand have identified several AIV subtypes in FGDs, including H10N6, H10N7, H11N6, H11N7, and H11N9 [4, 7, 8]. Serological evidence of antibodies against AIV-H5 has also been found in FGDs during a nationwide survey in 2012 [9]. Although Thailand has not reported any AI outbreaks in birds or humans since 2008, recent incidents of HPAI and LPAI in poultry and humans have occurred in neighboring countries such as Cambodia, Laos, and Vietnam, highlighting the ongoing regional risk [10–14].

Knowledge, attitude, and practice (KAP) studies are commonly used to evaluate a population’s understanding, perceptions, and behaviors related to specific health issues [15]. These studies typically use standardized, structured questionnaires administered during interviews [16] and offer valuable insights to enhance disease prevention and control efforts. Previous KAP surveys in China showed insufficient influenza knowledge among older and less educated poultry farmers, while studies in Taiwan found limited risk awareness and preventive practices among poultry workers following H5N2 outbreaks [17]. In Thailand, KAP studies have explored influenza-related awareness among Thai–Myanmar border populations during the H1N1 pandemic [18] and risk perceptions of AI among poultry farmers and traders in border regions with Laos [19]. Additional research has assessed farmers’ knowledge and practices related to poultry production systems [20].

Despite extensive virological surveillance and outbreak investigations of AI in Thailand, most existing research has concentrated on virus detection, molecular characterization, and ecological risk factors in poultry and wild birds. Although FGDs are widely recognized as key reservoirs and amplifiers of AIVs, especially in Southeast Asia, little attention has been paid to the human behavioral aspects related to FGD production systems. In Thailand, previous KAP studies have mainly focused on general poultry farmers, traders, border populations, or communities affected by specific influenza outbreaks, such as the H1N1 pandemic. These studies offer valuable insights but do not sufficiently address the unique occupational, ecological, and management features of FGD systems, which involve frequent duck movement, close human–animal contact, and shared environments with wild birds.

Furthermore, no comprehensive KAP assessment has been conducted specifically among individuals directly involved in FGD farming, trading, and related activities in central Thailand, a region with historically high FGD densities and past outbreaks of HPAI. The lack of recent AI outbreaks in Thailand since 2008 may also lead to risk complacency, which could affect preventive behaviors and biosecurity compliance among FGD stakeholders. Additionally, limited evidence exists on how demographic factors, educational level, vaccination history, animal contact patterns, and production practices impact AI-related knowledge, attitudes, and preventive behaviors within this high-risk group.

Given these gaps, the current study aimed to systematically evaluate levels of KAP regarding AI among people involved in FGD production in central Thailand. Specifically, the study intended to (i) measure AI-related knowledge, attitudes, and preventive behaviors among FGD farmers and their workers; (ii) identify socio-demographic, occupational, and exposure factors linked to differences in KAP levels; and (iii) explore the connections between knowledge, attitudes, and practices to better understand behavioral factors affecting AI prevention and control. By providing context-specific evidence from a high-risk yet understudied group, this research seeks to guide targeted risk communication, biosecurity measures, and One Health–based policies to improve AI prevention and preparedness in Thailand.

## MATERIALS AND METHODS

### Ethical approval

The study protocol was reviewed and approved by the Human Research Ethics Review Board of Chulalongkorn University, Thailand (IRB No. 152.1/63). All procedures were conducted in accordance with the ethical principles outlined in the Declaration of Helsinki. Before participation, all individuals were informed about the study objectives, procedures, the voluntary nature of participation, and their right to withdraw at any time without consequences. Written informed consent was obtained from all participants and, where applicable, from their legal guardians. Participant confidentiality was strictly maintained by anonymizing all data, securely storing records, and restricting access to authorized personnel only. Data were used exclusively for research purposes. Although no direct benefits were provided to participants, the findings are expected to contribute to a better understanding of AI-related KAPs, thereby supporting disease prevention and control efforts.

### Study period and location

An analytical cross-sectional study was carried out from January to May 2023 to evaluate AI-related KAP among individuals involved in FGD production systems. This study design was chosen for its efficiency in measuring KAP prevalence at a single point and for identifying links between KAP outcomes and related factors. The approach was cost-effective, time-efficient, and appropriate for targeted population assessments.

**Figure 1:**
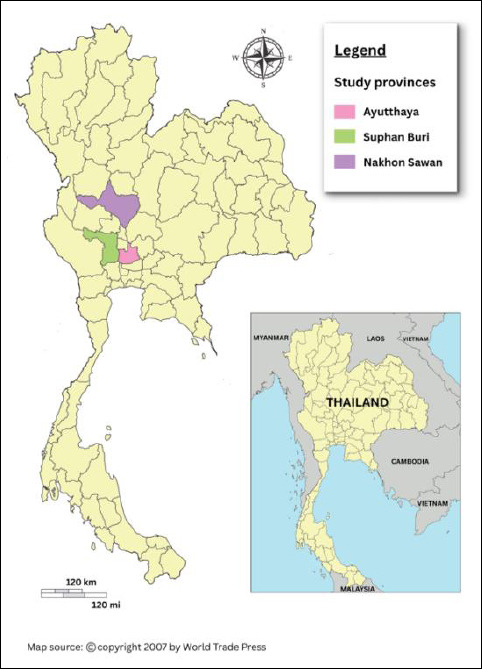
Map of three provinces in central Thailand for the knowledge, attitude, and practice study on avian influenza in free-grazing duck farmers.

The study was conducted in three provinces in central Thailand: Ayutthaya, Nakhon Sawan, and Suphan Buri (Figure 1). These provinces were chosen because they have a high density of FGD flocks, especially in Suphan Buri, where rice farming provides plentiful feeding grounds. The areas also serve as shared habitats for wild birds, increasing the risk of cross-species transmission of AIV. Importantly, outbreaks of AI were reported in backyard poultry, commercial farms, and FGDs in these provinces between 2004 and 2008. Site selection was further validated by historical outbreak data, ongoing FGD activity, and cooperation from farmers and local authorities.

### Study population and eligibility criteria

A total of 101 participants from Ayutthaya, Nakhon Sawan, and Suphan Buri provinces volunteered for the study. The target population included individuals involved in FGD farming, trading, and breeding, who were considered to have frequent human–animal interactions. Eligible participants needed to have been involved in the FGD production system and in contact with FGDs for at least three months before data collection to ensure sufficient experience and exposure.

Inclusion criteria were: (i) Thai nationality; (ii) age between 20 and 75 years; and (iii) willingness to participate and provide informed consent. Exclusion criteria included individuals who were unwilling to complete the study or who were unable to communicate effectively due to substance use or mental health conditions.

### Sample size determination and sampling method

The sample size was determined using a standard formula for estimating population proportions, assuming an estimated proportion (p) of 0.5 to maximize variance, a 95% confidence level (CI), and a margin of error (Δ) of 0.1. Based on these parameters, the minimum required sample size was 96 participants. To account for potential non-response or missing data, the sample size was increased to over 100 participants.

Participants were selected using simple random sampling from lists of registered FGD farmers provided by the Department of Livestock Development, Ministry of Agriculture and Cooperatives. Recruitment was facilitated through local authorities in the study areas, and all selected individuals agreed to participate.

### Questionnaire development and validation

Data were gathered using a structured questionnaire designed to evaluate AI-related KAPs. The questionnaire was created based on relevant literature and then reviewed and validated by three subject-matter experts to ensure content accuracy and consistency with the study objectives [21–23]. The Index of Item-Objective Congruence score was 0.98, showing excellent content validity.

Internal consistency reliability was evaluated using Cronbach’s alpha, resulting in values of 0.78 for knowledge (95% CI: 0.72–0.84), 0.51 for attitude (95% CI: 0.37–0.65), and 0.74 for practice (95% CI: 0.67–0.81). The lower reliability score for the attitude section was due to the limited number and diversity of items.

The questionnaire included 60 items divided into six sections: (i) socio-demographic features; (ii) contact with animals and exposure; (iii) health condition; (iv) knowledge of AI; (v) attitudes and perceptions about AI; and (vi) preventive behaviors and practices. The knowledge part contained 12 questions about clinical signs, transmission methods, and prevention strategies. The attitude part included 10 items on hygiene, food handling, and risk perception, while the practice part included 11 items focused on preventive actions such as handwashing, mask-wearing, respiratory hygiene, and seeking healthcare. The questionnaire was pretested with FGD farmers outside the study group, and their feedback was used to improve the tool before data collection.

### Data collection procedures

Data were gathered through face-to-face interviews to accommodate different literacy levels and ensure precise responses. Interviews were carried out by trained field enumerators, including master’s and Ph.D. students, at participants’ workplaces. The enumerators received two days of training from experienced researchers on interview techniques, ethical considerations, and cultural sensitivity. Field notes and observation checklists were used to enhance data accuracy. The principal investigator and senior researchers supervised all field activities. Data were checked for completeness and consistency before entry, anonymized, and organized using a codebook prepared by a statistician. No translation was needed, as all participants communicated in the central Thai dialect.

### Statistical analysis

Descriptive statistics, including frequencies, percentages, means, medians, and standard deviations, were used to summarize participant characteristics and KAP scores. Knowledge scores were categorized as good (≥80%), moderate (60%–79%), or poor (<60%) based on established criteria [24]. For regression analyses, total knowledge scores were converted to percentages and treated as continuous variables [25].

Attitude and practice scores were calculated as average values across their respective items, with response scales ranging from 0 to 5. Negative items were reverse-coded before analysis. Inferential analyses were performed in two stages. First, simple linear regression was used to identify associations between independent variables and KAP outcomes. Variables with p-values <0.2 were then included in multiple linear regression models using a backward stepwise approach, with statistical significance set at p <0.05.

Pearson’s correlation coefficients were calculated to evaluate the relationships between KAP scores. All analyses were conducted using R statistical software version 4.4.1 [26]. Model assumptions, including normality of residuals, homoscedasticity, and multicollinearity, were checked; variance inflation factor values ≤2 indicated acceptable collinearity. Interaction terms were tested but did not significantly enhance the model fit. A small number of outliers were removed to improve residual normality; however, some minor variance remained.

## RESULTS

### Characteristics of study participants

A total of 101 participants consented to join the study, including 73 FGD farmers (72.4%), 25 traders (24.7%), 2 students (1.9%), and one truck driver (1.0%). Participants were recruited from three provinces in central Thailand: Ayutthaya, Nakhon Sawan, and Suphan Buri. Of these, 60 (59.4%) were male and 41 (40.6%) were female. The average age was 48.68 ± 10.08 years, with most participants aged 41–50 years (37.6%) and 51–60 years (30.7%) (Table 1).

**Table 1:** Demographic information of study participants (N = 101).

Demographic information of the participants	Number (Percentage)
Gender	
Male	60 (59.4)
Female	41 (40.6)
Age	
< 30	3 (3.0)
31-40	16 (15.8)
41-50	38 (37.6)
51-60	31 (30.7)
> 60	13 (12.9)
The highest educational level	
No education	1 (1.0)
Primary school	63 (62.4)
Junior high school	25 (24.8)
High school	6 (5.9)
Vocational Certificate/High Vocational Certificate	2 (2.0)
Bachelor’s Degrees	4 (3.9)
Occupation	
Farmer	73 (72.4)
Trader	25 (24.7)
Transporter	1 (1.0)
Other	2 (1.9)
Type of raised FGD	
1 day old – start laying	27 (26.7)
1 day old – slaughtered (female)	1 (1.0)
1 day old – slaughtered (male)	1 (1.0)
Start laying—slaughtered	69 (68.3)
No duck	3 (3.0)
Main responsibility	
Take care of the duck hatching	2 (2.0)
Raise FGD	97 (96.0)
Buy FGD from the farmer	2 (2.0)
Number of years involved with FGD	
< 1	2 (2.0)
1-5	26 (25.7)
6-10	30 (29.7)
> 10	43 (42.6)
Vaccination	
No vaccination	6 (5.9)
Influenza vaccine	22 (21.8)
COVID-19	73 (72.3)

Data are presented as number (percentage). FGD = Free-grazing duck. Participants could report more than one type of vaccination; therefore, vaccination categories are not mutually exclusive.

Regarding educational attainment, most participants had completed primary education (62.4%), followed by junior high school (24.8%), high school (5.9%), vocational training (2.0%), and a bachelor’s degree (3.9%). The majority were directly involved as FGD farmers, with smaller numbers working as traders, transporters, or students. Concerning FGD production systems, 69 participants (68.3%) raised ducks from the start of laying until slaughter, while 27 (26.7%) raised ducks from 1 day old to the start of laying. Most participants (96.0%) reported being directly responsible for raising and managing FGDs. The length of involvement in the FGD business varied, with 42.6% reporting more than 10 years of experience, followed by 6–10 years (29.7%) and 1–5 years (25.7%) (Table 1).

### Influenza vaccination and general health information

Among the study participants, 73 (73.74%) reported receiving at least one vaccination while working with poultry. The majority had received the COVID-19 vaccine (69.31%), while only 25 participants (24.75%) reported getting an influenza vaccine. Of those vaccinated against influenza, just six participants (5.94%) received the vaccine annually, whereas 19 (18.81%) did not. Most influenza vaccinations were administered through local public health agencies (20.79%), while a smaller number of participants arranged and paid for their own vaccinations (2.97%). Influenza vaccination services were mainly accessed at local health facilities, followed by hospitals and workplace or private clinics (Table S1).

### Exposure to FGDs and other animals

Participants reported a mean duration of FGD involvement of 145.8 months (12.15 ± 11.44 years) and spent an average of 10.03 ± 6.27 hours per day working with FGDs. In the 12 months prior to the study, 38.61% of participants reported contact exclusively with FGDs, while 61.39% had contact with other domestic animals. Among those with additional animal contact, dogs were the most frequently reported (47.52%), followed by cats and chickens (both 19.80%). Contact frequency varied, with 23.76% of participants reporting daily contact.

Most participants (96.04%) reported that FGDs were not housed with other domestic animals. Only four participants reported mixed housing with dogs or birds (Supplementary Table 2).

### Overall KAP scores related to AI

The overall AI-related KAP scores are summarized in Table 2. The average knowledge score was 8.65 ± 2.39 out of 12, with a range of 3 to 11. The average attitude score was 3.63 ± 0.36 out of 5, while the average practice score was 3.17 ± 0.38 out of 5.

**Table 2:** Means, SD, minimum, maximum, and correlation of avian influenza-related knowledge, attitudes, and practices scores among study participants (n = 101).

KAP score	n	Min.	Max.	Mean ± SD	Median	IQR
The AI-related knowledge score	101	3.00	11.00	8.65 ± 2.39	9.00	(7, 11)
AI-related attitudes score	101	2.40	4.60	3.63 ± 0.36	3.60	(3.50, 3.83)
Score of AI-related practices	101	2.00	4.09	3.17 ± 0.38	3.27	(3.00, 3.44)

**Variable(s)**	**Knowledge**		**Attitude**		**Practice**	

Knowledge	1		-		-	
Attitude	0.20*		1		-	
Practice	0.11		0.40*		1	

Data are presented as mean ± standard deviation (SD). IQR = Interquartile range. Correlation coefficients are Pearson’s correlation coefficients.

* Correlation is statistically significant at p < 0.05 (two-tailed). Knowledge, attitude, and practice scores were treated as continuous variables.

Correlation analysis showed a moderate positive relationship between attitude and practice scores (r = 0.40, p < 0.05) and a weaker, yet significant, link between knowledge and attitude scores (r = 0.20, p < 0.05). There was no significant relationship between knowledge and practice scores (r = 0.11, p > 0.05). Most participants exhibited good knowledge (58.4%) and positive attitudes (66.3%) toward AI, while 38.6% demonstrated good preventive practices (Table 3). No significant gender differences were found across KAP levels.

**Table 3:** Frequency distribution of the avian influenza-related knowledge, attitudes, and practices scores among the study participants (N = 101).

AI-related knowledge score group	K score (0-100%)	Frequency (%)
Good	75%–100%	59 (58.4)
Moderate	50%–74%	29 (28.7)
Poor	<50%	13 (12.9)

**AI-related attitudes score group**	**A score (1-5)**	**Frequency (%)**

Good	3.6–5.00	67 (66.3)
Moderate	3.1–3.5	31 (30.7)
Poor	<3	3 (3.0)

**AI-related practices score group**	**P score (1-5)**	**Frequency (%)**

Good	3.3–5.00	39 (38.6)
Moderate	3.1–3.2	37 (36.6)
Poor	<3	25 (24.8)

K score was calculated as (knowledge score/12 × 100). Cut-off points for knowledge were defined as good (75%–100%), moderate (50%–74%), and poor (<50%). Attitude and practice scores represent mean item scores on a 1–5 scale. Cut-off points for attitude were defined as good (≥3.6), moderate (3.1–3.5), and poor (<3). Cut-off points for practice were defined as good (≥3.3), moderate (3.1–3.2), and poor (<3). Data are presented as number (percentage).

### Knowledge of AI

AI-related knowledge was evaluated using 12 items (K1–K12). Overall, 72.11% of responses were correct, with a median knowledge score of 9 out of 12 (75%), indicating a good level of understanding. The highest correct response rates were observed for questions about AIV pathogenicity (K1), distinguishing between avian and human influenza viruses (K5), and the risks associated with undercooked poultry products (K11). Conversely, knowledge about handwashing with soap as a preventive measure against AIV was significantly low, with 97.03% of participants answering incorrectly (Table 4).

**Table 4:** Factors influencing avian influenza-related knowledge among study participants.

	AI-related knowledge among participants	Frequency (%)	

		Correct answer	Incorrect answer
K.1	AIVs can be highly or low-pathogenic strains	96 (95.05)	5 (4.95)
K.2	AIV-infected ducks show no or mild clinical signs	65 (64.36)	36 (35.64)
K.3	AIV can be transmitted from avian to human	68 (67.33)	33 (32.67)
K.4	AIV can be transmitted from an avian to other animals	76 (75.25)	25 (24.75)
K.5	AIVs are not the same as influenza viruses in humans	93 (92.08)	8 (7.92)
K.6	Clinical signs of AIV infection include sudden death, respiratory distress, swollen head, and cyanosis of the comb and wattle.	86 (85.15)	15 (14.85)
K.7	AIV-infected avian may show clinical signs	79 (78.22)	22 (21.78)
K.8	Clinical signs of AIV infection include high fever, cough, muscle or body aches, shortness of breath or difficulty breathing, pneumonia, and/or death.	77 (76.24)	24 (23.76)
K.9	Humans can infect AIV from infected avian by contacting the body or secreting saliva, nasal discharge, or feces.	70 (69.31)	31 (30.69)
K.10	Humans can infect AIV by contacting the body or secreting saliva, nasal discharge, or stool.	67 (66.34)	34 (33.66)
K.11	Undercooked chicken or eggs can cause AIV infection	94 (93.07)	7 (6.93)
K.12	Frequent hand washing with soap can prevent AIV from occurring	3 (2.97)	98 (97.03)

Data are presented as number (percentage). K1–K12 represent individual knowledge items included in the questionnaire. Correct and incorrect responses were classified based on predefined answer keys validated by subject-matter experts. AIV = Avian influenza virus.

Multiple linear regression analysis identified four factors significantly linked to higher knowledge scores: fewer daily working hours with FGDs (β = −0.04, p = 0.03), contact with pet birds in the past year (β = 5.40, p = 0.03), history of vaccination while working with poultry (β = 2.46, p < 0.01), and self-managed influenza vaccination (β = 4.45, p < 0.01) (Table 5; Supplementary Table 3).

**Table 5:** Multiple linear regression analysis of factors influencing avian influenza-related knowledge, attitudes, and practices scores among participants.

Factors	Adjusted Coefficient	p-value	p-value

	(95% CI)	(t-test)	(F-test)
Knowledge (n = 99)			
Hours per day that you work with FGD (hours)	–0.37 (-0.71, -0.04)	0.03*	0.03*
Kind of animals that you have ever been involved, raised, or contacted during the past year (Pet bird)			0.03*
No (Ref.)	-	-	
Yes	44.98 (4.36, 85.6)	0.03*	
Ever been vaccinated while working with poultry?			<0.01*
No (Ref.)	-	-	
Yes	20.52 (12.82, 28.21)	<0.01*	
Who provided or arranged the influenza vaccination for you			<0.01*
Never been vaccinated	-	-	
Yourself	–37.06 (–61.55, –12.57)	<0.01*	
Local public health organizations	9.47 (0.58, 18.36)	0.04*	
Adjusted R^2^: 0.31, F: 9.86, α =0.05, *Statistically significant at p < 0.05			
Attitude (n = 101)			
The highest educational level		<0.01*	
No education (Ref)	-	-	
Primary school or below	16.9 (5.25, 28.54)	<0.01*	
Junior high school	18.24 (6.49, 29.99)	<0.01*	
High school	22.81 (10.46, 35.15)	<0.01*	
Vocational/High Vocational Certificate	14.5 (0.32, 28.68)	0.045*	
Bachelor’s Degrees	32.94 (18.76, 47.13)	<0.01*	
Occupation			<0.01*
Trader	-	-	
Farmer	6.39 (3.65, 9.13)	<0.01*	
Others	3.05 (–3.18, 9.28)	0.33	
Student	4.06 (–7.63, 15.74)	0.49	
Transporter	4.53 (–7.11, 16.18)	0.44	
Multiple R^2^: 0.44, F: 7.93, α =0.05, *Statistically significant at p < 0.05			
Practice (n = 99)			
The highest educational level			0.01*
No education (Ref)	-	-	
Primary school or below	3.16 (–11.27, 17.6)	0.66	
Junior high school	6.41 (–8.18, 21.01)	0.39	
High school	8.83 (–6.33, 23.98)	0.25	
Vocational/High Vocational Certificate	4.55 (–12.45, 21.55)	0.6	
Bachelor’s Degrees	16.42 (0.88, 31.95)	0.04*	
Kind of animals have you ever been involved, raised, or contacted during the past year? (Dog)			0.02*
No (ref.)	-	-	
Yes	3.42 (0.53,6.31)	0.02*	
Have you ever been vaccinated while working with poultry?			<0.01*
No (Ref.)	-	-	
Yes	5.89 (2.21, 9.57)	<0.01*	

Values are adjusted regression coefficients with 95% confidence intervals (CI). Variables with p < 0.20 in simple linear regression were entered into multiple linear regression models using a backward stepwise approach. Reference categories (Ref.) are indicated for categorical variables. Model fit is presented as adjusted R² and F-statistic. Statistical significance was set at p < 0.05. FGD = Free-grazing duck.

### Attitudes toward AI

Attitudes toward AI were evaluated using 10 items (A1–A10). Overall, 72.89% of responses indicated positive attitudes, with a median attitude score of 3.6 out of 5. The highest-rated statements involved handwashing after FGD contact, cleaning equipment and work areas, and avoiding the consumption or sale of dead ducks (Table 6).

**Table 6:** Factors influencing avian influenza-related attitudes among participants.

	AI-related attitudes among participants	Number (Percentage)						Mean ± SD	Attitude Level

		Do not know/did not answer	Strongly disagree	Disagree	Uncertain	Agree	Strongly agree		
A.1	Do you agree that AI is a highly contagious disease to humans?	9 (8.91)	5 (4.95)	14 (13.86)	15 (14.85)	52 (51.49)	6 (5.94)	3.44 ± 1.01	Agree
A.2	Do you agree that those who contact the FGD are at risk of AIV infection?	10 (9.90)	6 (5.94)	16 (15.84)	37 (36.63)	29 (28.71)	3 (2.97)	3.07 ± 0.97	Uncertain
A.3	Do you think that raising FGD in a farm system can prevent AIV transmission more effectively than a free-grazing system?	10 (9.90)	14 (13.86)	24 (23.76)	27 (26.73)	24 (23.76)	2 (1.98)	2.74 ± 1.08	Uncertain
A.4	Do you think that moving FGDs from one area to another increases the spread of AIV?	-	6 (5.94)	14 (13.86)	40 (39.60)	34 (33.66)	7 (6.93)	3.22 ± 0.98	Uncertain
A.5	Do you agree that you should wear gloves, masks, and boots when working with FGD to prevent AIV infection?	-	-	11 (10.89)	3 (2.97)	73 (72.28)	14 (13.86)	3.89 ± 0.77	Agree
A.6	Do you think that wearing gloves, masks, or boots while working makes it more difficult for you to work?"	1 (0.99)	5 (4.95)	16 (15.84)	19 (18.81)	55 (54.46)	5 (4.95)	3.39 ± 0.98	Agree
A.7	Do you agree that your hands should be washed with soap after touching FGD?	-	-	1 (0.99)	2 (1.98)	66 (65.35)	32 (31.68)	4.28 ± 0.55	Strongly agree
A.8	Do you agree that you should frequently clean the equipment and areas in contact with FGD or its secretions after work?	-	3 (2.97)	6 (5.94)	5 (4.95)	70 (69.31)	17 (16.83)	3.91 ± 0.85	Agree
A.9	Do you agree that unusual dead FGD should not be eaten or sold to others?	3 (2.97)	1 (0.99)	3 (2.97)	2 (1.98)	50 (49.50)	42 (41.58)	4.32 ± 0.75	Strongly agree
A.10	If you have a high fever with muscle ache, sore throat, and cough, do you agree that you should see a doctor immediately or within 24 h?	-	-	3 (2.97)	29 (28.71)	49 (48.51)	20 (19.80)	3.85 ± 0.77	Agree

Data are presented as number (percentage) and mean ± standard deviation (SD). Attitude items (A.1–A.10) were assessed using a 5-point Likert scale (1 = strongly disagree, 2 = disagree, 3 = uncertain, 4 = agree, 5 = strongly agree). Higher mean scores indicate more positive attitudes toward avian influenza prevention. Attitude levels were interpreted based on the mean item score. AI = Avian influenza, AIV = Avian influenza virus, FGD = Free-grazing duck.

Regression analysis showed that educational level and occupation were significantly linked to AI-related attitudes. Participants with higher levels of education, especially those with a bachelor’s degree, had more positive attitudes. Additionally, FGD farmers had significantly more positive attitudes than traders (Table 5; Supplementary Table 4).

### Practices related to AI prevention

AI-related practices were evaluated using 11 items (P1–P11). About 40.0% of responses reflected proactive behaviors, while 41.0% showed infrequent or no preventive practices. The median practice score was 3.27 out of 5. The top practices included handwashing after FGD contact, wearing surgical masks, and consistently washing hands during work activities (Table 7).

**Table 7:** Factors influencing avian influenza-related practices among study participants.

	AI-related practices among the study participants	Number (Percentage)						Mean ± SD	Practice level

		Do not know/did not answer	Never (0%)	Rarely (25%)	Sometimes (50%)	Often (75%)	Always (100%)		
P.1	Do you see a doctor immediately or within 24 h after experiencing high fever with muscle ache, sore throat, and cough?	1 (0.99)	1 (0.99)	8 (7.92)	50 (49.50)	21 (20.79)	20 (19.80)	3.51 ± 0.94	Often
P.2	Do you wash your hands with soap after contact or work with FGD?	-	1 (0.99)	3 (2.97)	17 (16.83)	51 (50.50)	29 (28.71)	4.03 ± 0.82	Often
P.3	Do you wear a surgical mask when contacting or working with the FGD?	-	16 (15.84)	3 (2.97)	10 (9.90)	39 (38.61)	33 (32.67)	3.69 ± 1.38	Often
P.4	Do you wear a plastic apron when contacting or working with the FGD?	-	52 (51.49)	41 (40.59)	6 (5.94)	2 (1.98)	-	1.58 ± 0.70	Never
P.5	Do you wear boots when contacting or working with the FGD?	-	15 (14.85)	8 (7.92)	12 (11.88)	51 (50.50)	15 (14.85)	3.43 ± 1.27	Often
P.6	Do you wash your hands with soap every time you come in contact with FGD?	-	-	4 (3.96)	23 (22.77)	46 (45.54)	28 (27.72)	3.97 ± 0.82	Often
P.7	Do you frequently clean the equipment or areas that come in contact with FGD or its secretions after use?	-	8 (7.92)	12 (11.88)	42 (41.58)	36 (35.64)	3 (2.97)	3.14 ± 0.95	Sometimes
P.8	Do you have a rest day for the hatchery, slaughterhouse, duck house, or areas that are in contact with FGD?	19 (9.90)	25 (24.75)	-	16 (15.84)	26 (25.74)	24 (23.76)	3.26 ± 1.55	Sometimes
P.9	Do you raise other animals with FGD or do you share the area with FGD?	-	88 (87.13)	6 (5.94)	1 (0.99)	3 (2.97)	3 (2.97)	1.29 ± 0.88	Never
P.10	If an FGD or AIV outbreak usually results in death, do you move your FGD flock to other areas?	8 (7.92)	41 (40.59)	23 (22.77)	14 (13.86)	6 (5.94)	9 (8.91)	2.13 ± 1.31	Rarely
P.11	If an FGD gets sick or dies from an unknown cause, you will eat or sell that FGD. ^N^	1 (0.99)	94 (93.07)	1 (0.99)	3 (2.97)	-	2 (1.98)	1.15 ± 0.66	Never

Data are presented as number (percentage) and mean ± standard deviation (SD). Practice items (P.1–P.11) were assessed using a 5-point Likert scale (0 = never, 1 = rarely, 2 = sometimes, 3 = often, 4 = always). Higher mean scores indicate more frequent adoption of recommended preventive practices. Practice levels were interpreted based on the mean item score. Item P.11 is a negatively worded question (N) and was reverse-coded before analysis. FGD = Free-grazing duck, AIV = Avian influenza virus.

Multiple linear regression analysis identified four factors significantly associated with better AI-related practices: higher educational level, involvement in FGD rearing from early production stages, contact with other animals during the past year, and a history of vaccination while working with poultry (Table 5; Supplementary Table 5).

## DISCUSSION

### Ecological and production significance of FGDs

FGDs are important reservoirs of AIVs. Their ecological uniqueness, including free-ranging behavior and frequent movement through rice fields, helps spread influenza viruses over large geographic areas. FGDs also share habitats with wild birds, which may facilitate AIV transmission and spread among different species. The economic importance of FGD systems is tied to a specific duck production method commonly used in Southeast Asia that aims to reduce feeding costs. In this system, FGDs are raised in harvested rice paddies to consume leftover rice grains, snails, and insects, thereby helping farmers with pest control [27]. According to national policy, FGDs in Thailand must be vaccinated against duck plague and fowl cholera; however, there is currently no policy on the use of AI vaccines in poultry.

### Overview of study findings

This study provides initial insights into the KAP regarding AI among farmers participating in FGDs in Thailand. From January to May 2023, 101 participants from Ayutthaya, Nakhon Sawan, and Suphan Buri provinces were surveyed. The participants included FGD farmers (72.4%), FGD traders (24.7%), truck drivers (1.0%), and others (1.9%). Most were male (59.4%) and aged 40–50 years (37.6%). The survey found that most participants possessed good knowledge of AI, with an average score of 8.65 out of 12. Participants also held positive attitudes toward AI (average score of 3.63 out of 5) and engaged in proactive preventive practices (average score of 3.17 out of 5).

### Factors associated with KAP

Several factors were notably associated with AI-related knowledge, including the hours spent daily working with FGDs, contact with other animals in the past year, vaccination while working with FGDs, and self-arranged influenza vaccination. Factors significantly linked to AI-related attitudes included participants’ education level and occupation. AI-related practices were heavily influenced by education level, the type of FGD conducted, contact with other animals, and vaccination during FGD activities. These results imply that vaccination history might affect both knowledge and practices and could serve as a predictor of KAP within the community.

Notably, participants showed limited knowledge about handwashing with soap as a way to prevent AI (2.96%). However, despite this knowledge gap, their attitudes and practices suggested they often washed their hands with soap after contact with FGDs. This finding contrasts with a previous study reporting that Thai residents in AI outbreak areas typically practiced handwashing after slaughtering or cooking meat but were less likely to do so during poultry contact or egg collection activities [28]. Certain confounding variables may influence the observed link between higher education levels and better preventive practices. For example, farmers with higher levels of education might have greater access to healthcare services and health information, which could lead to higher KAP scores.

### Comparison with previous KAP studies

This study shows that FGD farmers in Thailand generally know about AI. The findings agree with earlier research among poultry farm workers in Indonesia, where most participants (91%) showed good knowledge of AI [18, 29]. Similar results have also been seen in studies measuring H1N1-related KAP among university students in South Korea and the United Kingdom [30, 31]. In contrast, the findings differ from a KAP study conducted with poultry farm workers in Guinea, which reported low levels of knowledge about AI (42.9%) [32]. The results also differ from a KAP study on H1N1 among communities along the Thai–Myanmar border, where knowledge levels were found to be low [18].

In the present study, FGD farmers who spent more hours working with FGDs demonstrated higher AI-related knowledge. Educational level was significantly associated with attitudes toward AI, consistent with findings from China, where participants in urban areas with higher levels of education exhibited greater AI awareness [33]. Notably, FGD farmers showed higher AI-related attitude scores compared with traders and transporters. Educational level was also a key factor influencing AI-related practices (p < 0.05). Farmers who raised FGDs from the duckling stage scored significantly higher on practice measures. Additionally, FGD farmers commonly wore masks and boots while working with FGDs and frequently washed their hands with soap afterward, a practice known to significantly reduce the risk of AI [34]. However, some preventive measures, such as wearing aprons and implementing flock resting periods, were rarely adopted and merit further research because of their potential to prevent disease transmission.

### Surveillance context and study limitations

Thailand maintains an active annual AI surveillance program carried out by government agencies, including the Department of Livestock Development, as well as by our research team. Both FGD flocks and farmers must register with the Department of Livestock Development (DLD), and official certification is required for flock movement. Despite this structured surveillance system, several limitations of the current study should be acknowledged.

First, the cross-sectional design limits the ability to establish causal relationships between KAP variables. Second, the non-normal distribution of KAP scores may have constrained differentiation among participants, although the results were interpreted cautiously. Third, participants were recruited solely from three central provinces of Thailand, which may limit the generalizability of the findings to other regions. Finally, recall and interview bias may have influenced the accuracy of self-reported past activities.

## CONCLUSION

This study offers the first comprehensive assessment of KAP related to AI among individuals involved in FGD production systems in Thailand. Overall, the findings show that most participants possessed good knowledge of AI, held positive attitudes toward disease prevention, and practiced preventive measures at a moderate level. The average knowledge score was 8.65 out of 12, while attitude and practice scores averaged 3.63 and 3.17 out of 5, respectively. Knowledge was significantly associated with daily working hours, FGDs, contact with other animals, and influenza vaccination history. Attitudes were mainly influenced by educational level and occupation, whereas preventive practices were affected by education, the type of FGD production system, animal contact, and vaccination while working with poultry. Although awareness of handwashing as a preventive measure was notably low, self-reported attitudes and practices indicated frequent handwashing after FGD contact.

These findings have important implications for AI prevention and One Health–based disease control strategies in Thailand. The observed associations between vaccination history and improved knowledge and practices highlight the potential role of vaccination programs as entry points for health education and risk communication. Targeted educational interventions focusing on practical biosecurity measures, particularly hand hygiene, use of personal protective equipment, and flock management, are warranted, especially for individuals with lower educational attainment. Strengthening coordination between veterinary and public health authorities can further improve surveillance, risk awareness, and adherence to preventive measures among FGD farmers, traders, and related workers.

A major strength of this study is its focus on a high-risk yet understudied population directly involved in FGD production systems. The use of a validated, structured questionnaire, face-to-face interviews, and multivariable statistical analyses enabled a detailed assessment of KAP levels and related factors. Additionally, the study offers context-specific evidence from central Thailand, a region historically linked to AI outbreaks and intensive FGD activity.

Several limitations should be acknowledged. First, the cross-sectional design prevents establishing causality between related factors and KAP outcomes. Second, KAP scores did not follow a normal distribution, which could have limited the ability to distinguish between participant groups. Third, the study was conducted in only three central provinces, which may limit the applicability of the findings to the entire country. Lastly, recall bias and social desirability bias might have affected self-reported responses.

Future studies should expand geographic coverage to include other regions of Thailand and neighboring countries where FGD systems are common. Longitudinal studies are recommended to evaluate changes in KAP over time and to assess the effectiveness of targeted interventions. Combining behavioral assessments with virological surveillance and environmental data would further improve One Health risk evaluations. Additionally, qualitative research could explore contextual barriers to adopting specific biosecurity practices, such as apron use and flock resting periods.

In conclusion, FGD farmers in Thailand generally have adequate knowledge and positive attitudes toward AI; however, gaps still exist in preventive practices. Addressing these gaps through targeted education, vaccination awareness programs, and strengthened One Health collaboration is crucial to reducing the risk of AI transmission at the human–animal–environment interface. The findings of this study provide valuable evidence to support policy development, surveillance improvements, and practical interventions to enhance AI preparedness and prevention in FGD production systems.

## DATA AVAILABILITY

The supplementary data can be made available from the corresponding author upon request.

## AUTHORS’ CONTRIBUTIONS

SB: Drafted the manuscript. SB, KC, and NB: Designed the questionnaire and prepared the data. SB, SC, CN, and KT: Performed the questionnaire interviews and statistical analysis. KT, CS, and KS: Participated in the statistical analysis. SP, KS, and AA: Designed the study, performed the data analysis, and drafted, revised, and approved the manuscript. All authors have read and approved the final version of the manuscript.
